# Type VI secretion systems at the interface between bacteria and eukaryotic cells

**DOI:** 10.1590/1678-4685-GMB-2026-0024

**Published:** 2026-09-14

**Authors:** Izabella Santos Mori Bragil, Bianca Bontempi Batista, José Felipe Teixeira da Silva Santos, Natália Carolina Drebes Dörr, Cristina Elisa Alvarez-Martinez

**Affiliations:** 1 Universidade Estadual de Campinas (UNICAMP), Instituto de Biologia, Departamento de Genética, Evolução, Microbiologia e Imunologia, Campinas, SP, Brazil.

**Keywords:** Effectors, bacteria, virulence, microbial competition, predation

## Abstract

Bacterial type VI secretion systems (T6SSs) are molecular devices that traverse the bacterial envelope and mediate the translocation of protein effectors directly into prokaryotic or eukaryotic cells, thereby interfering with diverse cellular processes in target organisms. T6SSs involved in interbacterial competition have been widely described and functionally characterized, together with a remarkable arsenal of cognate toxins. In contrast, comparatively less is known about anti-eukaryotic T6SSs. This review summarizes recent advances in the study of T6SSs that target eukaryotic cells, highlighting their roles in virulence, resistance to environmental predators, and microbial competition. We discuss T6SS-mediated interactions with protozoan predators, fungi, animal hosts and plants, emphasizing the expanding repertoire of T6SS effectors that modulate conserved eukaryotic pathways. Together, these findings reveal the ecological and pathogenic relevance of T6SS-driven trans-kingdom interactions.

## Introduction

Type Six Secretion Systems (T6SS) are nanomachines used by many Gram-negative (diderm) bacteria to translocate toxic protein effectors directly into other cells, playing important roles in bacterial environmental adaptation and colonization of distinct niches. T6SS effectors usually target conserved prokaryotic or eukaryotic cell functions, such as membranes, nucleic acids, peptidoglycan, or the eukaryotic cytoskeleton (Allsopp and Bernal, 2023; Wohlfarth et al., 2026). While the T6SS was initially discovered as an anti-eukaryotic weapon, most T6SSs described to date are involved in interbacterial competition, revealing a new mechanism used by bacteria for interference competition. Furthermore, recent evidence has expanded the realm of T6SS functions to include fungal killing and metal ion scavenging, the latter involving the secretion of proteins into the extracellular milieu (Allsopp and Bernal, 2023; Trunk et al., 2018). 

T6SSs clusters are widespread in Pseudomonadota and are formed by 13-14 conserved genes that encode the structural components of the machinery, in addition to a varied repertoire of accessory genes whose products are involved in effector recruitment and regulation. T6SSs have been classified into four evolutionary divergent subtypes, named i-iv, which differ in their structural components and assembly mechanisms. Besides the canonical T6SS^i^ subtype widely studied in Pseudomonadota, subtypes ii and iii are found in *Francisella* species and members of the Bacteroidetes phylum, respectively, and all of them are thought to have evolved from phage-derived structures. Finally, T6SS^iv^ is more distantly related to T6SS^i-iii^ and closer to extracellular contractile injection systems, described in *Amoebophilus asiaticus* (Russell et al., 2014; Böck et al., 2017; Wohlfarth et al., 2026). 

Subtype i T6SS has been further classified into six clades (i1, i2, i3, i4a, i4b and i5) based on phylogenetic analyses of core components (Boyer et al., 2009). Typical T6SS^i^ machines resemble inverted bacteriophage tails and are composed of three main portions: (i) a membrane complex that spans both outer and inner membranes; (ii) a tail formed by an inner Hcp tube surrounded by a contractile sheath and tipped by VgrG and PAAR proteins; and (iii) a baseplate that anchors the tail and connects it to the membrane complex. Sheath contraction allows the tube to be propelled out of the cell and to puncture prokaryotic or eukaryotic recipient cells, translocating associated protein effectors. Translocation of T6SS effectors occurs through two mechanisms: either as specialized domains fused to the C-terminal regions of contractile tube components VgrG, PAAR, and Hcp (specialized effectors), or via non-covalent interactions with Hcp, VgrG, or PAAR (cargo effectors), which may be indirectly mediated by adaptor proteins. Identification of T6SS cargo effectors may be challenging due to the absence of a conserved recruitment motif and often relies on their genetic linkage to *paar, vgrG,* or *hcp* genes. However, several recruitment motifs have been recently identified in subsets of T6SS effectors, including MIX, RIX, PIX, and WHIX domains (Allsopp and Bernal, 2023). 

**Figure 1 -:**
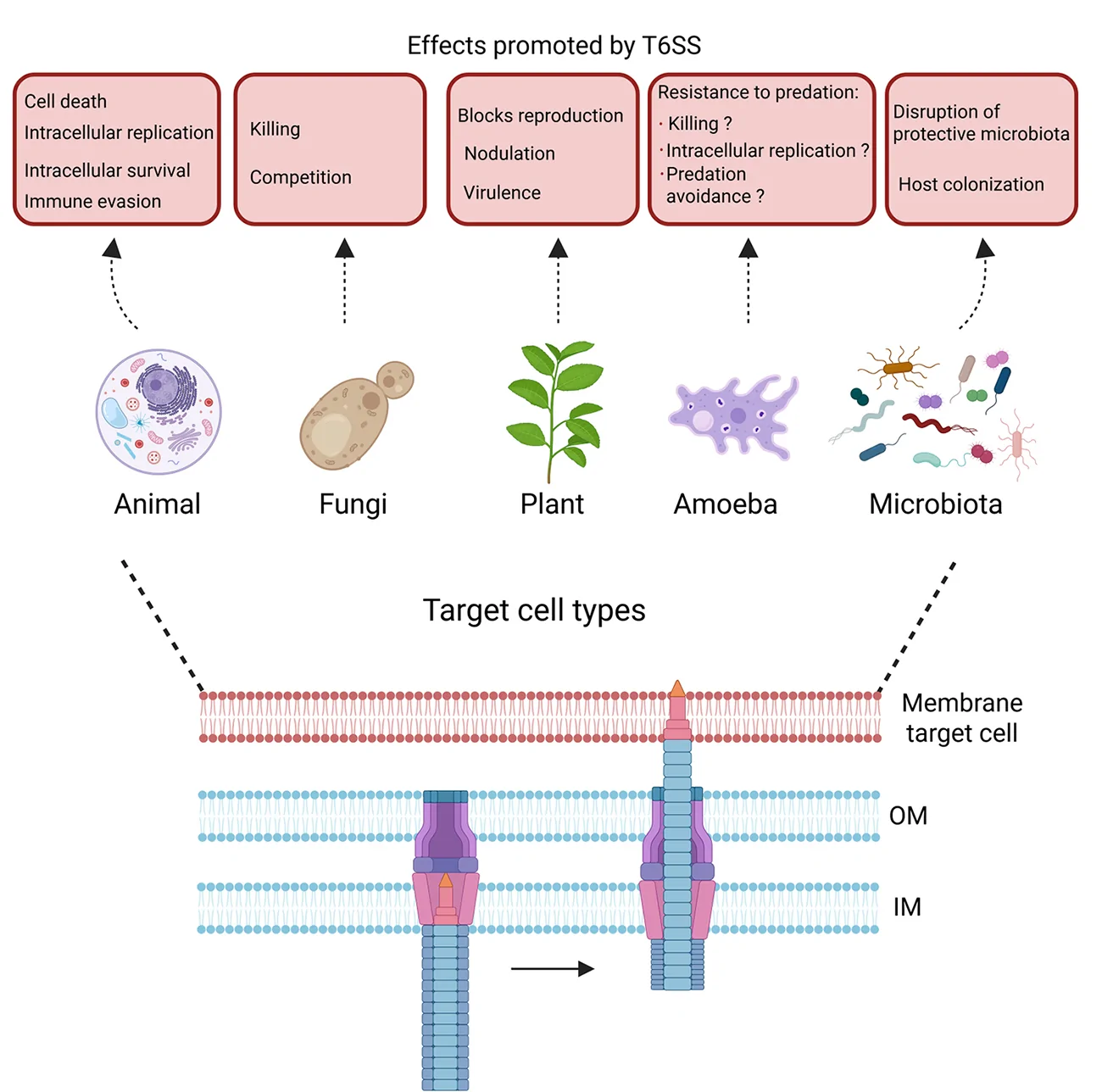
Effects of the T6SS in bacterial interactions with eukaryotic cells. The T6SS is a dynamic bacterial nanomachine that enables microorganisms to deliver effector proteins either into the extracellular environment or directly into target cells. This versatile machinery can act on a wide range of cell types and may act as an important virulence factor during infection. When targeting animal cells, T6SS effectors may contribute to immune evasion, intracellular survival, intracellular replication, or induction of cell death. T6SSs may also affect bacteria-plant interactions, but the direct translocation of T6SS effectors to plant cells has yet to be demonstrated. T6SSs also target other eukaryotic organisms, including fungi and amoebae, thereby promoting competition and resistance to predation, respectively. Prokaryotic cells are likewise susceptible to T6SS attacks, which may indirectly affect virulence by targeting the host microbiota to enhance colonization and establish a competitive advantage.

**Figure 2-:**
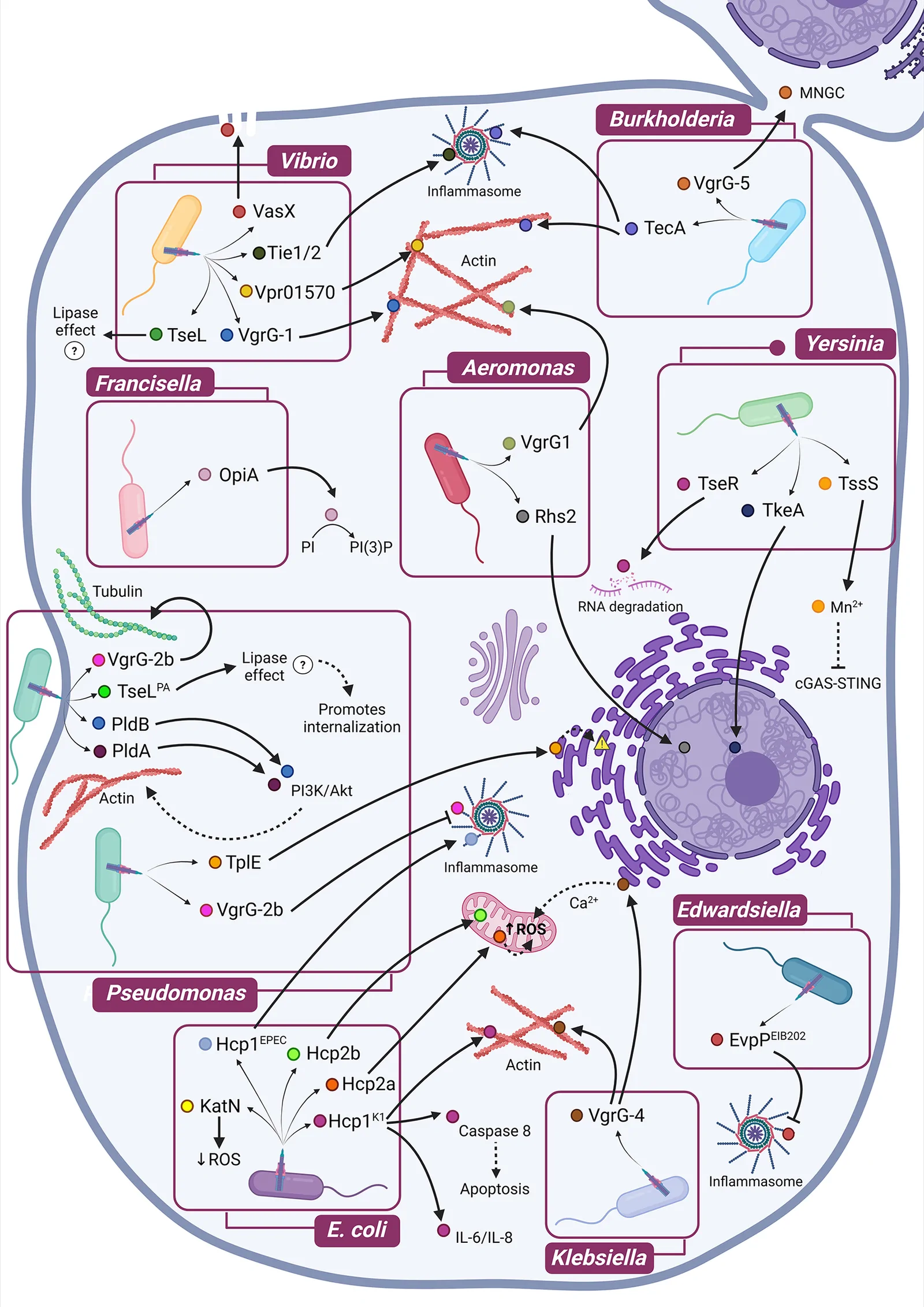
Anti-eukaryotic type VI secretion system (T6SS) effectors and their mechanisms of action. The schematic depicts representative anti-eukaryotic effectors and their reported or proposed activities. In *Vibrio*, VasX forms membrane pores, TseL exhibits putative lipase activity, VgrG-1 promotes actin crosslinking, Tie1/2 are required for inflammasome-mediated cell death and Vpr01570 promotes actin rearrangement. In *Francisella*, OpiA manipulates phosphoinositide metabolism. In *Aeromonas*, VgrG1 disrupts the host actin cytoskeleton and Rhs2 functions as a DNase. In *Burkholderia*, TecA deamidates Rho GTPases, leading to actin cytoskeleton disruption and inflammasome activation, while VgrG-5 induces cell fusion and multinucleated giant cell formation (MNGC). In *Yersinia*, TssS chelates Mn²⁺ to suppress cGAS-STING signaling, while TseR promotes RNA degradation and TkeA causes DNA damage. In *Pseudomonas*, PldA and PldB are phospholipases that activate PI3K/Akt signaling for the subsequent actin rearrangement and protrusion formation, TseL^PA^ displays lipase-like activity to promote internalization, and TplE disrupts endoplasmic reticulum membranes, while VgrG2b interacts with γ-tubulin complexes to promote internalization and inhibits NLRP3 inflammasome activation. In *E. coli*, Hcp1^EPEC^ activates the NLRP3 inflammasome and induces cell death, Hcp1^K1^ induces actin rearrangement, initiates caspase-8-dependent apoptosis and stimulates the release of pro-inflammatory cytokines such as IL-6 and IL8, KatN reduces ROS through Mn-dependent catalase activity, and Hcp2a/Hcp2b impair mitochondrial function. In *Klebsiella*, VgrG-4 promotes Ca²⁺ transfer from the endoplasmic reticulum to mitochondria, causing ROS accumulation and inhibiting inflammatory responses mediated by NFκB, in addition to a role in cytoskeleton rearrangements in phagocytic cells. In *Edwardsiella tarda* strain EIB202, EvpP functions by inhibiting NLRP3 inflammasome activation in macrophages through interference with Ca²⁺- and Jnk-mediated signaling, which supports bacterial persistence and colonization. Collectively, these effectors act on membranes, cytoskeletal structures, signaling pathways, nucleic acids, mitochondria, the inflammasome, and the endoplasmic reticulum, illustrating the breadth of T6SS-mediated anti-eukaryotic strategies.

**Figure 3 -:**
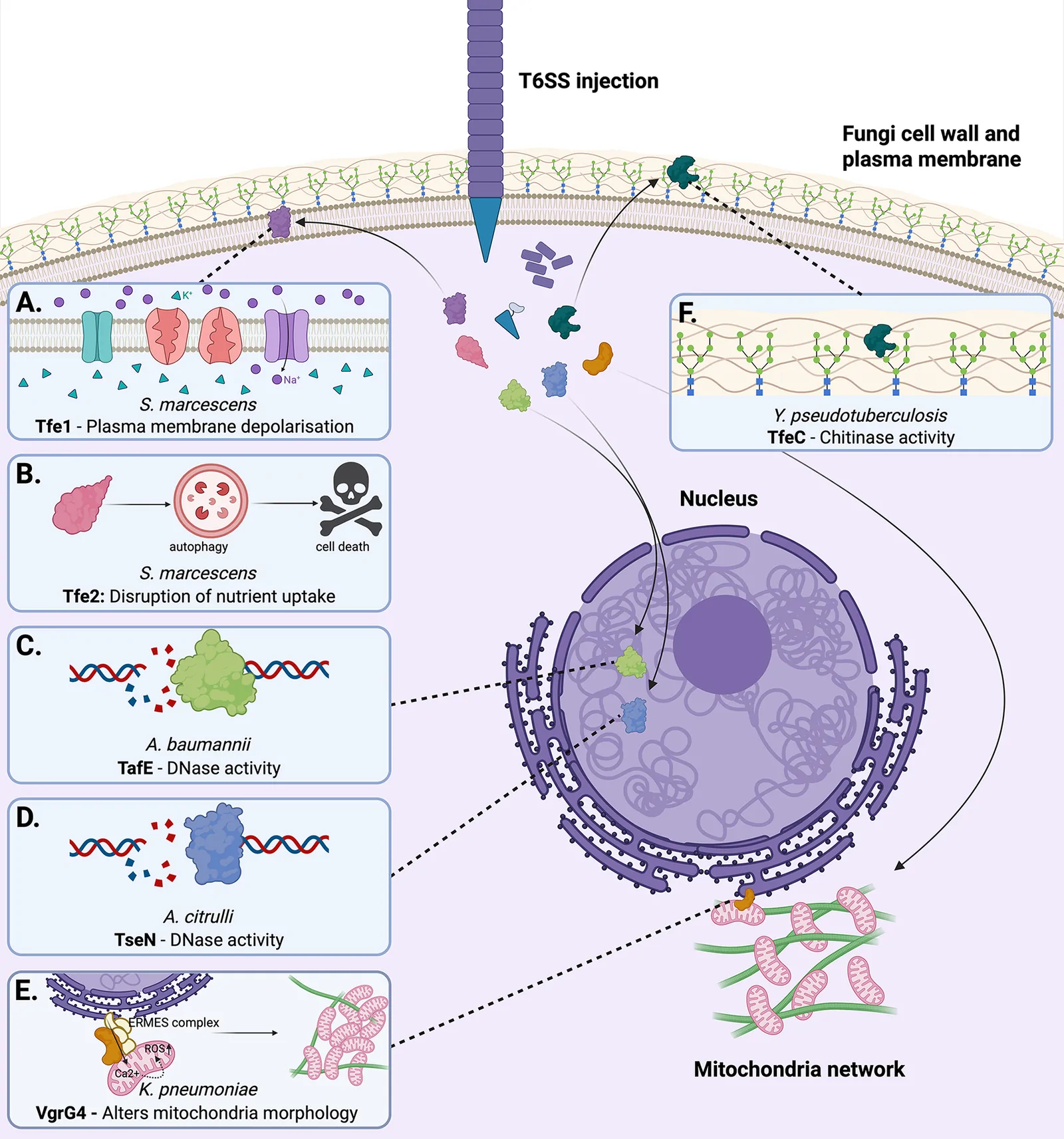
T6SS-delivered antifungal effectors. **A)** *S. marcescens* Tfe1 promotes membrane depolarization, leading to loss of membrane integrity; **B)** Tfe2 is secreted by *S. marcescens* T6SS and impacts the membrane transporter activity of *Candida spp.*, leading to metabolic imbalance and ultimately inducing autophagy; **C)** The *A. baumannii* TafE promotes bacterial and fungal killing and is a DNase that is translocated into the yeast cell nucleus but cannot cross the HeLa cell nucleolar compartment; **D)** *A. citrulli* TseN is a novel DNase with yeast nuclear localization sequences; **E)** *K. pneumoniae* VgrG4 localizes with the *S. cerevisiae* ERMES (“Endoplasmic Reticulum and Mitochondria encounter structures”) complex in the outer mitochondrial membrane. This enhances Ca^2+^ exchange between the ER and mitochondria, leading to mitochondrial network disruption; **F)** TfeC is a new chitinase secreted by the *Y. pseudotuberculosis* T6SS4 that disrupts the cell wall. The structures depicted in the figure are not to scale. Effectors with unknown cellular targets were not shown.

T6SSs have evolved as versatile nanomachines with multiple functions, including overcoming bacterial competitors, eukaryotic predators, and host defenses (Hachani et al., 2016). Significant progress in understanding the relevance of the T6SS in interbacterial competition has been made since the first reports in the early 2000s. In contrast, the role of T6SSs in interactions with eukaryotes remains a much less explored yet exciting field, despite several key advances in recent years. In this review, we describe T6SS clusters that play important roles in bacterial interactions with eukaryotic cells, with a focus on those that exert direct effects on eukaryotic hosts, predators, or competitors. Importantly, while the four T6SS subtypes are named using Roman numerals, different T6SS clusters found in the same genome can be named using Arabic numerals. This nomenclature will be used throughout the manuscript, following the original studies in which these clusters were described. An overview of the diverse eukaryotic targets of T6SSs, along with their respective effects, is presented in Figure 1 and Table 1. Anti-eukaryotic T6SS effectors are summarized in Table S1 and Figures 2 and 3.

**Table 1-:** T6SSs that target eukaryotic cells.

Cluster name	Bacteria	T6SS classification^1^	Target organism	Mechanism
**T6SS involved in competition with fungi**				
T6SS	*A. baumannii*	i4b	*E. coli*; yeast cells	Killing
T6SS	*A. citrulli*	i3	*S. cerevisiae*, *C. neoformans* and *Candida* species; Gram-positive and Gram-negative bacteria	Killing
T6SS-I	K. pneumoniae*	i2	*C. albicans* and *S. cerevisiae*	Killing
T6SS	*L. enzymogenes*		Yeast cells; filamentous fungi	Killing
T6SS	*M. xanthus*	i1	Phytopathogenic fungi	Killing
T6SS	*P. syringae* pv. Tomato DC3000	i1	Eukaryotic microbes (amoebae and yeast)	N. D.
T6SS	*P. fluorescens*		Fungi	Killing
T6SS4	*Y. pseudotuberculosis*		Fungi	Killing
T6SS	*S. marcescens*		Yeast cells; filamentous fungi	Killing
**T6SS involved in resistance to predation**				
T6SS	*A. hydrophila* AH54		*D. discoideum*	N. D.
T6SS	*P. chlororaphis*		Amoebae, protozoa and nematodes	N. D.
T6SS	*Paraburkolderia bonniea*		*D. discoideum*	Symbiont transmission
T6SS	*X. citri* pv. *citri* 306	i3	*D. discoideum*	N. D.
T6SS	*V. cholerae*	i1	*D. discoideum*	N.D.
**T6SS involved in virulence**				
T6SS	*A. dhakensis*	i1	*D. discoideum*; mouse	Immune evasion
T6SS	*A. veronii* TH0426	i1	Zebrafish	N. D.
T6SS1	Avian Pathogenic *E. coli*	i2	Ducks	Internalization
T6SS2	Avian Pathogenic *E. coli*	i1	Ducks	Internalization
T6SS-5	*B. thailandensis*	i1	Human cells	Intracellular survival
T6SS	*B. cenocepacia*	i4	Macrophages	N. D.
T6SS-4	*B. glumae* BGR1	i2	Plant	N. D.
T6SS-5	*B. glumae* BGR1	Unknown	Plant	N. D.
T6SS-1	*B. mallei*	i1	Macrophages	Internalization
T6SS-1	*B. pseudomallei* K96243	i1	Macrophages	Intracellular survival
T6SS	*E. coli* W24		Mouse	Pyroptosis
T6SS	*E. coli* K1		HBMEC	Internalization
T6SS	Enterohemorrhagic*E. coli*		BALB/c mice	Intracellular survival
T6SS	*E. tarda*	i4b	C57BL/6 mice	Immune evasion
T6SS	*E. ictaluri*	i4	Fish	N. D.
T6SS-I	K. pneumoniae*	i2	*G. mellonella;* yeast competition	Immune evasion
T6SS	*F. tularensis*	i2	Mice; *G. mellonella*	Intracellular survival
T6SS-1	*P. ananatis* str DZ-12 and LMG2665	i3	Plant	Colonization
T6SS	*P. protegens* CHA0	i3	*Pieris brassicae* (insect)	Internalization
T6SS	*P. plecoglossicida*		*Epinephelus coioides* (fish)	N. D.
T6SS-II	*P. aeruginosa* UCBPP-PA14	i1	*Arabidopsis thaliana* (plant) and mouse	Internalization
T6SS1	*V. fischerii*		Zebrafish and *Artemia nauplii*	N.D.
T6SS	*V. coralliilyticus*		*Artemia salina*	N.D.
T6SS3	*V. proteolyticus*		*Artemia salina* (shrimp)	Pyroptosis
T6SS-3	*Y. pseudotuberculosis*	i3	BALB/c mice	Colonization
T6SS-4	*Y. pseudotuberculosis*		C57BL/6 mice	N.D.
T6SS-3	*X. oryzae* pv. *oryzae* PXO99A	i3	Plant	N. D.

N.D. - Not determined; HBMEC - Human brain microvascular endothelial cells; *The T6SS-I of *K. pneumoniae* is involved in both competition and virulence. 1- according to the SecReT6 database (SecReT6, T6SS database).

## T6SS in environmental interactions: Defense against protozoan predation and competition with fungi 

A growing body of evidence in host-pathogen research supports the notion that microbial virulence traits may have originated from environmental selective pressures, such as iron limitation, oxidative stress, and predation by amoebae and nematodes (Dunn et al., 2017; Hoque et al., 2022). Many environmental bacteria resist predation by secreting effector proteins that either promote predator avoidance, enable bacterial intracellular replication or kill amoebae. The role of T6SS as a weapon against amoeba predation has been described in some bacterial species, including *Vibrio cholerae*, *Xanthomonas citri* and *Aeromonas hydrophila* (Figure 1, Table 1) (Bayer-Santos et al., 2018; Drebes Dörr and Blokesch, 2020; Wang et al., 2025a). 

*V. cholerae* is an aquatic bacterium that inhabits marine and estuarine environments and is the etiological agent of the acute diarrheal disease cholera, with different strains showing variable levels of virulence to humans. The primarily antibacterial T6SS of *V. cholerae* is also required for resistance to predation by the soil amoeba *Dictyostelium discoideum* in a subset of isolates, relying mainly on an evolved effector with actin crosslinking activity (Pukatzki et al., 2007; Drebes Dörr and Blokesch, 2020). Interestingly, this T6SS-mediated anti-grazing activity was not detected in pandemic strains, in which the T6SS is silent under conditions of co-incubation with amoeba cells (Drebes Dörr and Blokesch, 2020). Intra-amoeba replication of *V. cholerae* has been demonstrated in the aquatic amoeba *Acanthamoeba castellanii*; however, in this case, the mechanism does not rely on an active T6SS (Van der Henst et al., 2018). A recent study also described a role for the antibacterial T6SS of a multidrug-resistant clinical isolate of the opportunistic pathogen *A. hydrophila* against grazing by *D. discoideum* (Wang et al., 2025a). *A. hydrophila* is an aquatic bacterium that causes infections in a variety of aquatic animals and is also a food-borne human pathogen associated with soft-tissue infections and diarrhea. These examples suggest that anti-grazing activity may be an important adaptation to natural and host environments. 

Plant-associated microbes, including both pathogens and plant growth-promoting bacteria (PGPB), also use their T6SS as defense against grazing by soil-dwelling eukaryotic predators. In the citrus plant pathogen *X. citri* pv. *citri*, the T6SS is required for resistance against *D. discoideum* predation and the T6SS gene cluster is specifically induced in response to incubation with amoeba (Bayer-Santos et al., 2018). Interestingly, *X. citri’s* T6SS is not required for virulence or competition against bacteria or fungi, suggesting a specific anti-amoebal function. A T6SS anti-amoebal activity has also been reported for the pathogen *Pseudomonas syringae* pv. *tomato*, which harbors two T6SS clusters. Loss of this phenotype was only observed when both clusters were inactivated, which also led to reduced bacterial competition and fungal killing, indicating a dual function against distinct eukaryotes and bacteria (Haapalainen et al., 2012). Similarly, inactivation of the two T6SSs of the beneficial PGPB *Pseudomonas chlororaphis* led to reduced toxicity against the soil predators *D. discoideum*, the ciliate *Tetrahymena thermophila* and the nematode *Caenorhabditis elegans*, also causing reduced fitness in the rhizosphere (Boak et al., 2022).

In many cases, amoebae act as reservoirs of microbes that infect humans, as exemplified by *Mycobacterium* species, *Legionella pneumophila*, and *V. cholerae* (Dunn et al., 2017). Despite these many examples of anti-amoeba activity, a role for the T6SS in establishing an intracellular niche-such as described for the T4SSs of *Legionella* and *Coxiella* and the ESX system of *Mycobacterium*-has not been demonstrated. Notably, T6SS+ species of *Paraburkholderia* can escape phagocytosis and stably colonize wild *D. discoideum* strains. A recent study showed that the T6SS is not required for a stable symbiosis but contributes to horizontal transmission to non-exposed amoeba hosts, suggesting a role in the maintenance of this bacteria-amoeba symbiosis in the environment (Chen et al., 2025). Future studies may reveal new mechanisms of bacteria-amoeba interactions mediated by T6SS.

A growing number of studies have revealed that antibacterial T6SSs may also target fungal cells, providing a fitness advantage against these eukaryotic competitors in diverse habitats (Figure 1, Table 1). A dual-function anti-fungal/anti-bacterial T6SS was first described in the opportunistic bacterium *Serratia marcescens*, where it antagonizes both yeast cells and filamentous fungi (Trunk et al., 2018). Following this initial report, antifungal activity of otherwise antibacterial T6SSs has been described in several bacterial species. One such example is the T6SS of the ESKAPE pathogen *Acinetobacter baumannii,* which was initially characterized for its bacterial killing activity against both Gram-negative and Gram-positive cells and has been recently shown to compete against *Saccharomyces cerevisiae* and the nosocomial pathogens *Candida albicans* and *Candida glabrata* (Luo et al., 2023). 

In a hypervirulent strain of the opportunistic pathogen *Klebsiella pneumoniae*, the T6SS-mediated microbial competition against bacteria and the yeast species *S. cerevisiae* and *C. albicans* is induced by several environmental cues, including antimicrobial peptides and acidic pH (Storey et al., 2020). Moreover, *K. pneumoniae*’s T6SS also targets human lung epithelial cells, indicating a role in virulence in this species (Sá-Pessoa et al., 2023). A recent study has uncovered a role for the *Yersinia pseudotuberculosis* T6SS4 cluster in fungal killing, in addition to its previously characterized functions in zinc transport and stress responses. This specific T6SS is sufficient to modulate fungal colonization in the mouse gut, reducing the abundance of members of the Ascomycota phylum, which includes *Candida* species (Yan et al., 2025; Zhu et al., 2026). Remarkably, *Y. pseudotuberculosis* induces its T6SS4 in response to fungi presence, by sensing the *C. albicans* quorum sensing molecule tyrosol via the two-component system EnvZ-OmpR, revealing a new interkingdom sensing pathway.

Antifungal T6SSs have also been identified in plant-associated and soil bacteria. In the plant pathogen of cucurbit crops *Acidovorax citrulli*, a potent T6SS with activity against Gram-negative and Gram-positive bacteria also promotes fungi killing, efficiently outcompeting *Pichia pastoris*, *S. cerevisiae*, and *C. albicans* (Pei et al., 2022). In the plant beneficial bacterium *Lysobacter enzymogenes* the T6SS mediates contact-dependent growth inhibition of a variety of plant pathogenic fungi, protecting plants against infection and modulating the fungal community in the soil (Lin et al., 2025). In contrast to most antifungal T6SSs reported to date, the T6SS from *L. enzymogenes* is not involved in bacterial antagonism; instead, this function is mediated by the T4SS in this bacterium (Lin *et al.,* 2025). A role for bacterial T6SSs in modulating fungal soil communities is also suggested by the antifungal activity of the T6SS of *Myxococcus xanthus*, a soil bacterium that preys on both bacteria and fungi (Liu et al., 2021). Likewise, T6SS-mediated antagonism against fungal cells has been demonstrated for the plant beneficial *Pseudomonas fluorescens* and the pathogen *P. syringae* pv. *tomato* (Haapalainen et al., 2012; Lin *et al.,* 2025). 

Collectively, these studies highlight that T6SSs are central mediators of bacterial-eukaryotic interactions across diverse natural environments, facilitating niche colonization and shaping microbiome composition.

## Establishing Infection: T6SS in Bacteria-Host Interactions

T6SS loci have been shown to be required for infection in animal models and eukaryotic cell cultures, supporting the notion that host subversion is another important function of this secretion system (Figure 1, Table 1). It is important to note, however, that the mechanisms underlying reduced virulence after T6SS inactivation remain uncharacterized in many cases. Such attenuation may stem from decreased bacterial fitness within polymicrobial communities rather than a direct contribution to host subversion or immune evasion. In this review, we focus on T6SSs that are directly involved in pathogenicity, either through manipulation of host defenses and/or invasion promotion.

*Francisella tularensis* is a highly virulent, facultative intracellular bacterium and the causative agent of tularemia in humans and animals, also infecting blood sucking arthropods that therefore function as vectors. Its primary replication site is phagocytes, but it may also replicate in non-phagocytic cells, such as erythrocytes. To replicate, *F. tularensis* must escape from the phagosome into the host cell cytoplasm, a process largely dependent on the *Francisella* Pathogenicity Island (FPI) (Degabriel et al., 2023). FPI encodes a set of proteins that constitute a subtype ii T6SS, which is essential for phagosomal escape, intracellular replication within macrophages and virulence in animal models, such as mice and *Galleria mellonella* (Barker et al., 2009; Bröms et al., 2010; Degabriel *et al.,* 2023). Beyond intramacrophage replication, Schmitt et al. (2017) demonstrated that the T6SS of *F. tularensis* can mediate invasion of red blood cells, a process relevant to tick colonization. 

The role of T6SS in bacterial invasion has also been demonstrated in the opportunistic pathogen *Pseudomonas aeruginosa*, which harbors three evolutionarily distinct T6SS clusters, each delivering a unique repertoire of effectors. Recently, a fourth T6SS has been identified in 1% of strains with publicly available genomes (Habich et al., 2025). While cluster H1-T6SS acts as a specialized defensive device mediating interbacterial competition, the H2-T6SS and H3-T6SS are predominantly associated with pathogenesis in different models, including plants, nematodes and mice lung and burn infection models, exhibiting some degree of functional redundancy (Lesic et al., 2009; Sana et al., 2012; Colautti et al., 2025). Notably, H2-T6SS also targets bacterial cells. H2-T6SS and H3-T6SS contribute to *P. aeruginosa* internalization into non-phagocytic epithelial cells by secreting distinct effectors that induce actin rearrangements and membrane protrusions (Sana *et al.,* 2012; Jiang et al., 2014; Swart et al., 2024). A recent study demonstrated that after T6SS-mediated invasion of goblet cells, the T3SS promotes host cell death, facilitating bacterial dissemination in the epithelial tissue (Sana *et al.,* 2012; Jiang *et al.,* 2014; Swart *et al.,* 2024). In phagocytic cells, H2-T6SS also contributes to pathogenesis by inhibiting inflammasome activation (Qian et al., 2025). A role for the T6SS in inhibiting host inflammasome signaling has also been demonstrated for the facultative intracellular bacterium *Edwardsiella tarda*, which requires the T6SS for efficient colonization *in vivo* (Chen et al., 2017).

A distinct mechanism of inflammasome manipulation is mediated by the T6SS-3 cluster of the marine pathogenic bacterium *Vibrio proteolyticus*. In this case, the T6SS is toxic to macrophages by induction of an inflammasome-mediated pyroptotic cell death. This process depends on bacterial internalization by phagocytosis and relies on two effectors, Tie1 and Tie2 (see below) (Cohen et al., 2022). Notably, the *V. proteolyticus* T6SS-3 is conserved across several other *Vibrio* species that are pathogenic to humans and marine animals (Cohen *et al.,* 2022). *V. proteolyticus* encodes two additional T6SS clusters, T6SS-1 and T6SS-2. The former is homologous to the T6SS of *V. cholerae* and exhibits both anti-eukaryotic and bactericidal activities (Ray et al., 2017). In this case, toxicity is mediated by cytoskeleton rearrangements in phagocytic cells upon internalization (Ray *et al.,* 2017). T6SS-mediated toxicity towards macrophage cells has also been demonstrated for the coral pathogen *V. coralliilyticus* (Mass et al., 2024). Nine anti-eukaryotic effectors have been described in this species, but the mechanisms involved in eukaryotic cytotoxicity remain elusive. Similarly, T6SS-1 of the bobtail squid symbiont *Vibrio fischeri* is involved in zebrafish and *Artemia* brine shrimp larvae mortality (Gaddy et al., 2025), suggesting a conserved function as an anti-eukaryotic T6SS in this species. 

*Burkholderia* species may encode up to six T6SS clusters. Among them, the T6SS-5 plays a key role in intracellular dissemination of *B. pseudomallei*, the causative agent of melioidosis, its surrogate model *B. thailandensis,* and the closely related species *B. mallei* (Schell et al., 2007; Burtnick et al., 2010 *,* 2011; Schwarz et al., 2014; Hopf et al., 2014). These species are facultative intracellular bacteria that invade both phagocytic and non-phagocytic cells and escape from the primary vacuole into the cytosol, where they have the unique ability to promote host cell fusions, generating multinucleated giant cells (MNGC), therefore enabling intercellular spread without exposure to the extracellular environment. In *B. pseudomallei,* the T6SS-5 is required for MNGC formation, intracellular replication and cytotoxicity to macrophages, and T6SS-5 mutant strains are attenuated in mice and the Syrian hamster model of melioidosis (Pilatz et al., 2006; Burtnick *et al.,* 2011; Hopf *et al.,* 2014). It has been recently shown that this T6SS is also involved in apoptotic cell death exacerbation in macrophages and in the lungs of infected mice (Stockton et al., 2024). Similarly, *B. thailandensis* and *B. mallei* use their T6SS-5 to induce cell-cell fusions and MNGC, and mutations that abolish T6SS-5 functionality resulted in reduced virulence in animal models (Burtnick *et al.,* 2010; Schwarz *et al.,* 2014). A recent study further demonstrated that *B. thailandensis* employs its T6SS-5 to lyse membrane protrusions, enabling direct cell-to-cell to spread (Plum et al., 2024). 

The T6SS is also required for pathogenesis of *B. cenocepacia*, an opportunistic pathogen that relies on intracellular infection of mucosal macrophages for colonization, causing chronic lung infection in cystic fibrosis patients (Aubert et al., 2008). In this species, the T6SS is essential for survival within macrophages, by subverting several mechanisms required for bacterial clearance. These mechanisms include actin cytoskeleton disruption and delayed maturation of the NADPH complex involved in oxidative burst in the phagocytic vacuole (Aubert *et al.,* 2008; Flannagan et al., 2012; Rosales-Reyes et al., 2012). 

Despite its absence in commensal *Escherichia coli*, the T6SS is found in pathotypes within the species. The avian pathogenic *E. coli* (APEC; strain TW-XM) encodes two T6SS clusters and both contribute to adherence and invasion of host cells, although their relative contribution to pathogenesis differ with respect to host cell specificity: the T6SS-2 is involved in direct interactions with brain cells, whilst T6SS-1 contributes to invasion and adhesion to different cell lines and is also bactericidal (Ma et al., 2014). Based on these findings, it was suggested that the T6SS1 contributes to APEC’s systemic proliferation, whereas the T6SS-2 plays a specific role in cerebral infection (Ma *et al.,* 2014). A similar role in adhesion and invasion of endothelial brain cells, in addition to effector-mediated toxicity, has been associated with the T6SS of a meningitis-causing *E. coli* K1 strain (Zhou et al., 2012). Finally, in the intestinal *E. coli* pathogroup (IPEC), an enterohemorrhagic strain (EHEC) deploys a T6SS for intramacrophage survival and is required for full virulence in a mouse model (Wan et al., 2017). 

The enteric pathogen *Yersinia pseudotuberculosis* (*Yptb*) possesses four T6SS clusters (T6SS-1 to T6SS-4), which are differentially regulated in response to temperature, suggesting distinct adaptive functions (Zhang et al., 2011). The T6SS-3 and T6SS-4 contribute to colonization of the gastrointestinal system and enhance survival of *Yptb* in infected mice. Notably, the T6SS-3 secretes DNase and RNase effectors that also have bactericidal activity, whilst T6SS-4 affects a eukaryotic-specific pathway, suppressing the innate immune response mediated by the STING pathway (Zhu et al., 2021). 

*Aeromonas dhakensis* encodes a T6SS that exhibits both anti-eukaryotic and antibacterial activities, contributing to mice colonization, resistance against phagocytosis by macrophages and predation by the amoeba *D. discoideum* (Suarez et al., 2008; Liang et al., 2021). 

In addition to T6SS clusters with direct effects in virulence described above, many bactericidal T6SSs indirectly contribute to animal host colonization through interference competition, i.e., by disrupting the commensal microbiota. Moreover, a few T6SSs that secrete nutrient scavenging proteins into the extracellular milieu may contribute to virulence by resource competition. A detailed description of these T6SSs is beyond the scope of this review, but we will briefly mention a few notable examples. In *Salmonella enterica* serovar Typhimurium a T6SS is required for early establishment within the host gut, providing competitive advantage against members of the microbiota (Sana et al., 2016). A similar effect of T6SS in host microbiota disruption has been demonstrated during mice colonization by *Aeromonas veronii* and insect larvae colonization by *Pseudomonas protegens* (Vacheron et al., 2019; Jiang et al., 2024)*.* A role for contact-independent T6SSs in virulence has been demonstrated in *B. thailandensis* and *Yptb*, which secrete metal scavenging effectors that contribute to host colonization (Wang et al., 2015; Si et al., 2017; Zhu et al., 2021). 

T6SSs are widely distributed among plant-associated bacteria. Indeed, the first phenotype reported for T6SS mutants was described in *Rhizobium leguminosarum*, where inactivation of the T6SS resulted in increased nodulation (Bladergroen et al., 2003). 

Since then, roles for T6SSs in nodulation, virulence and seed transmission have been described based on phenotypes of T6SS deleted strains (reviewed by Mijatović Scouten et al., 2025). Nevertheless, a direct effect inside plant cells resulting from effector delivery remains to be demonstrated, raising the question as to whether T6SS can directly target plant cells for virulence, as shown for other bacterial secretion systems. Instead, deleterious effects of T6SS inactivation on virulence of phytopathogenic bacteria may be indirect, resulting from reduced fitness in bacterial competition in the plant environment (reviewed by Mijatović Scouten *et al.,* 2025). Notably, some of these species encode different T6SS gene clusters, suggesting specialized roles. *Burkholderia glumae* strain BGR1 encodes four T6SS clusters, with T6SS-4 and T6SS-5 contributing to virulence in rice plants, whilst its T6SS-1 is bactericidal (Kim et al., 2020). Similarly, comparative genome analyses of *Pantoea ananatis* strains identified three putative gene clusters, named T6SS-1 to T6SS-3. T6SS-1 and T6SS-3 are present in all analyzed strains, whereas the T6SS-2 is found only in a subset of them (Shyntum et al., 2014). T6SS-1 is required for virulence and bacterial competition in onion plants. Additionally, a T6SS effector that is toxic when expressed in yeast has been identified, suggesting a potential role in interactions with host cells (see below) (Shyntum *et al.,* 2015; Zhao et al., 2024; Martinkus et al., 2025). *Xanthomonas oryzae* pv*. oryzae* causes bacterial blight in rice, one of the most destructive diseases affecting this crop. Recently, it was shown that *X. oryzae* pv. *oryzae* PXO99 infects flowers of the nonhost plant *Arabidopsis* and suppresses ovule initiation, reducing seed number without causing visible disease symptoms. This phenomenon is caused by a phospholipase effector (TleB) of a T6SS that also displays bactericidal activity (T6SS-2), suggesting it may be also directly targeted to the plant cell (Yang et al., 2025). 

Altogether, these findings highlight the importance of identifying and characterizing effectors that directly target eukaryotic cellular processes for a better understanding of the mechanisms by which T6SSs impact bacterial virulence and host interaction. 

## T6SS effectors targeting eukaryotic cells

In this section, we briefly describe effectors with known activity against eukaryotic cells and involved in host colonization, as well as fungal competition. For clarity, they are presented separately for each bacterial group and summarized in Table S1. Intracellular targets and molecular mechanisms underlying each effector’s activity are illustrated in Figure 2 for effectors targeting host cells and in Figure 3 for those involved in antifungal activity.

### T6SS effectors targeting eukaryotic cells for host manipulation

*Francisella species*

T6SS substrates encoded both within and outside the FPI were identified by a proteome-wide secretion assay (Eshraghi et al., 2016). FPI-encoded effectors are designated as “pathogenicity determinant proteins” (Pdp) and include PdpC and PdpD, while those encoded outside the FPI were designated as “outside pathogenicity island” (Opi) proteins: OpiA, OpiB-1, and OpiB-3, which were all shown to contribute to intracellular growth in THP-1 macrophages (Eshraghi *et al.,* 2016). Further studies demonstrated that OpiA from *F. novicida* functions as a phosphatidylinositol 3-kinase (PI3K)-like enzyme and modifies host endosomal phosphoinositides, delaying endosomal maturation and promoting bacterial escape from the phagosome into the cytoplasm (Figure 2) (Ledvina et al., 2018). OpiA was not required for invasion or replication in non-phagocytic cells, including erythrocytes, in a live vaccine strain of *F. tularensis*. However, Δ*opiA* strains presented elevated TNF-α levels, suggesting a role for this effector in modulation of host immune responses (Cantlay et al., 2020). OpiA was also important for pathogenesis in the chicken embryo tularemia model, indicating broader roles in infection beyond the macrophage environment (Cantlay *et al.,* 2020). 

Effectors PdpC and PdpD are required for phagosome escape and intramacrophage replication, inflammasome activation and pathogenicity in mice (Brodmann et al., 2017; Long et al., 2012; Uda et al., 2014). Phenotypic analyses of single and double mutants of these effectors indicate that their effects are additive, though PdpC exhibits a more prominent role (Brodmann *et al.,* 2017). PdpC is also essential for erythrocyte invasion, representing the first known T6SS effector directly implicated in red blood cell infection (Cantlay et al., 2022). Further studies may reveal the biological function of PdpC and PdpD during interaction with host cells.

*Pseudomonas aeruginosa*

Comparative genomics showed that *P. aeruginosa* strains possess both core and accessory effectors associated with their three major T6SS clusters (H1-, H2-, and H3-T6SS). This distribution suggests that core effectors mediate functions shared across strains in diverse environments, whereas accessory effectors confer selective advantages to certain strains in specific ecological niches (Habich et al., 2025). 

VgrG2b is a H2-T6SS accessory effector and is part of the most abundant combined effector set (CES), which includes 21 proteins found in over 100 strains (Habich et al., 2025). VgrG2b is an evolved VgrG with a C-terminal extension containing a putative zinc-dependent metallopeptidase domain (PS00142). Initially, it was reported to be delivered into host cells by the H2-T6SS, contributing to the microtubule-dependent internalization of *P. aeruginosa* into non-phagocytic cells through interaction with the γ-tubulin ring complex (Sana et al., 2015). A more recent study demonstrated that VgrG2b also inhibits inflammasome activation in macrophages, facilitating chronic infections (Figure 2). VgrG2b is recognized and cleaved by caspase-11, generating a free C-terminal effector fragment that competes with NEK7 for binding to NLRP3, leading to inhibition of NLRP3 inflammasome activation (Qian et al., 2025). Notably, VgrG2b cleavage is required for infection in mice, further demonstrating the relevance of this effector to *P. aeruginosa* virulence (Qian *et al.,* 2025).

A superfamily of bacterial phospholipase/ lipase enzymes has been characterized as T6SS-associated lipase effectors (Tle), which were divided into five families, Tle 1-5 (Russell et al., 2013). These effectors employ diverse biochemical strategies and collectively are capable of degrading phospholipids at multiple chemical linkages, ultimately disrupting target cell membranes. Such lipases are particularly abundant within the *P. aeruginosa* H2-T6SS effector arsenal, which has evolved to act against both bacterial and eukaryotic cells. Among these, the Tle5 family trans-kingdom effectors PldA and PldB are accessory and core substrates, respectively (Russell *et al.,* 2013; Jiang et al., 2014; Wettstadt et al., 2019; Habich et al., 2025). In eukaryotic cells, PldA and PldB activate the PI3K/Akt signaling pathway upon binding to Akt1/2 kinases, subsequently leading to actin rearrangement and protrusion formation, enabling the internalization of *P. aeruginosa* into the host cell (Figure 2) (Jiang *et al.,* 2014). 

TplE is a core H2-T6SS effector that belongs to the Tle4 lipase family and acts both against bacteria and eukaryotic cells. Notably, TplE contains a PGAP1-like (post-glycosylphosphatidylinositol attachment to proteins 1) domain, which is found in eukaryotic serine hydrolases that are associated with the endoplasmic reticulum (ER) (Figure 2). *In vivo* experiments demonstrated that TplE is translocated into mammalian cells by the H2-T6SS and localizes to the ER, where its phospholipase activity causes ER disruption, ultimately inducing the unfolded protein response and autophagy (Jiang et al., 2016).

A widely distributed lipase effector from the Tle2 family was recently characterized in a clinical *P. aeruginosa* strain from a COVID-19 patient (strain LYSZa7), which showed homology to *V. cholerae* TseL (see below) (Ren et al., 2023). TseL^PA^ is bactericidal and contributes to invasion of host cells, suggesting its role as a trans-kindgom effector (Figure 2) (Ren *et al.,* 2023). However, further studies are required to understand its role during infection by *P. aeruginosa*.

*Yersinia pseudotuberculosis*

The effectors TseR and TkeA, both secreted by the T6SS-3 cluster of *Yptb*, function as trans-kingdom nucleases capable of targeting both eukaryotic and bacterial cells. TseR is a specialized effector, with a C-terminal Ntox44 (novel toxin) domain combined with an N-terminal PAAR domain. Experiments with HeLa cells revealed that this effector is cytotoxic and acts as a divalent cation-dependent ribonuclease that preferentially cleaves single-stranded RNA (Figure 2) (Wang et al., 2025 b ). TkeA, in turn, is a DNase that induces DNA breaks when translocated to the nucleus of HeLa and Caco2 cells (Figure 2). The fragmented genetic material subsequently leaks into the cytoplasm, activating the cyclic GMP-AMP synthase (cGAS)/ stimulator of interferon genes (STING) pathway and triggering a TNF-mediated apoptotic pathway. In parallel, TkeA targets competing bacteria by degrading their genomes, thereby enhancing the competitive advantage of *Yptb* in the intestinal environment (Song et al., 2025). 

In addition to the nuclease effectors of T6SS-3, *Yptb* also deploys TssS, a 48-amino acid micropeptide primarily secreted by the T6SS-4, which promotes virulence via a distinct molecular mechanism. TssS functions as a highly specific Mn²⁺ chelator, sequestering this immunostimulatory metal ion inside host cells. Infection by *Yptb* induces the release of Mn²⁺ from intracellular organelles, which would normally amplify cGAS-STING activation and therefore work as a host defense. However, upon T6SS-dependent translocation into the host cytosol, TssS sequesters Mn²⁺, thereby blocking Mn²⁺-enhanced STING oligomerization and downstream innate immune signaling (Figure 2) (Zhu et al., 2021).

*Vibrio species*

Several effectors mediating anti-eukaryotic activity have been characterized in *Vibrio* species, including VgrG-1, TseL, VasX, Vpr01570, Tie1 and Tie2 (Figure 2). *V. cholerae*’s effectors VgrG-1, TseL and VasX are required for virulence against the amoeba *D. discoideum*. VgrG-1 is a specialized effector that contains a 446-amino acid C-terminal extension homologous to the actin cross-linking domain (ACD) of the *V. cholerae* RtxA toxin (Pukatzki et al., 2007). Experiments using cytoplasmic extracts of *D. discoideum* and macrophages combined with *in vivo* assays in J774 cells demonstrated that the ACD of VgrG-1 mediates actin cross-linking, leading to cytoskeletal disorganization and cell rounding (Pukatzki *et al.,* 2006 *,* 2007). This activity is essential for T6SS-dependent cytotoxicity, indicating that VgrG-1 acts exclusively in the intoxication of eukaryotic cells and does not contribute to interbacterial competition. In contrast, TseL and VasX contribute to both eukaryotic cell intoxication and antibacterial activity. TseL is a phospholipase effector whose activity and cytotoxicity depends on a conserved catalytic residue (Asp425), demonstrating that phospholipid hydrolysis is critical for its function (Dong et al., 2013). VasX, in turn, contains an N-terminal Pleckstrin-homology (PH) domain that mediates interactions with eukaryotic membrane phospholipids, and a C-terminal colicin-like domain (Miyata et al., 2011; Liang et al., 2019). This structural organization enables VasX to associate with host membranes and promote pore formation, ultimately compromising cell integrity (Figure 2). The combined action of TseL and VasX have been demonstrated in experiments using *D. discoideum*, where single deletion of either *tseL* or *vasX* reduced *V. cholerae* virulence against amoebae, while double deletion completely abolished killing, resulting in a phenotype equivalent to that of a T6SS-null mutant (Dong *et al.,* 2013). Notably, this loss of virulence did not impair VgrG-1 ACD secretion or activity, indicating that TseL and VasX act at cytotoxic stages distinct from and complementary to cytoskeletal disruption (Dong *et al.,* 2013).

In *V. proteolyticus*, the effectors Tie1 (T6SS3 inflammasome-inducing effector 1) and Tie2 are both secreted by the T6SS-3 cluster and are required for inflammasome-mediated cell death in macrophages (Figure 2). However, the molecular mechanisms underlying inflammasome activation remain unclear (Cohen et al., 2022). Another *V. proteolyticus* anti-eukaryotic effector, Vpr01570, contains a cytotoxic necrotizing factor 1 (CNF1) deamidase domain that is predicted to activate Rho GTPases, which have a key role in cytoskeleton organization (Ray et al., 2017). Vpr01570 is transported by the T6SS-1 into macrophages and induces the formation of actin ruffles, a phenomenon dependent on the catalytic activity of the CNF1 domain (Figure 2). Ectopic expression of Vpr01570 in macrophages was sufficient to induce actin ruffling but failed to reproduce the cell-spreading phenotype observed during infection, indicating that additional T6SS effectors cooperate to produce the full cytopathic effect (Ray *et al.,* 2017).

*Klebsiella pneumoniae*

Hypervirulent strains of *K. pneumoniae*, such as Kp52145, encode a functional T6SS that contributes to inter-bacterial competition, anti-fungal activity, and host colonization (Sá-Pessoa et al., 2023). The T6SS effector VgrG4 contains a C-terminal DUF2345 that promotes accumulation of reactive oxygen species (ROS) in bacterial and yeast cells, which was therefore named ROS-toxic domain (RTD) (Storey et al., 2020). Further studies showed that VgrG4 exerts anti-eukaryotic activity by targeting mitochondrial homeostasis. VgrG4 colocalizes with the ER protein mitofusin 2 (Mfn2) and promotes Ca²⁺ transfer from the ER to mitochondria, leading to activation of the mitochondrial fission regulator Drp1 and fragmentation of the mitochondrial network (Sá-Pessoa *et al.,* 2023). This Ca²⁺ influx also activates the mitochondrial innate immunity receptor NLRX1, which induces ROS production, ultimately inhibiting NF-κB-mediated inflammatory response (Figure 2). In yeast, the C-terminal RTD of VgrG4 is sufficient to induce mitochondrial condensation and oxidative stress, while the N-terminal VgrG domain mediates localization to ER-mitochondria contact sites (Sá-Pessoa *et al.,* 2023). Additionally, the C-terminal domain of VgrG4 directly binds actin and promotes cytoskeletal remodeling in phagocytic cells (do Nascimento Soares et al., 2022).

*Escherichia coli*

In the meningitis-causing *E. coli* K1 strain, Hcp1 is a T6SS-secreted effector that disrupts the physiology of human brain microvascular endothelial cells. Hcp1 induces actin cytoskeleton rearrangement, triggers caspase-8-dependent apoptosis and stimulates the release of pro-inflammatory cytokines such as IL-6 and IL-8, thereby promoting blood-brain barrier inflammation and contributing to the pathogenesis of meningitis (Figure 2) (Zhou et al., 2012). 

In *E. coli* EPEC, the T6SS effector Hcp1 enhances bacterial virulence by inducing inflammation and cell death in host tissues. Hcp1 promotes acute liver injury (ALI) and acute kidney injury (AKI) in infected mice. Moreover, Hcp1 triggers pyroptosis in renal tubular epithelial cells by activating the NLRP3 inflammasome. This leads to caspase-1-mediated cleavage of gasdermin D (GSDMD), generating GSDMD-N, which forms membrane pores and promotes the release of active IL-1β, ultimately causing inflammatory cell death and amplifying tissue damage (Wang et al., 2023). 

In avian pathogenic *E. coli* (APEC), two major variants, Hcp2a and Hcp2b, have been characterized for their capacity to disrupt mitochondrial function in avian DF-1 cells (Lu et al., 2024; Lu *et al.,* 2025). Hcp2a localizes to mitochondria through its interaction with LETM1, a mitochondrial inner membrane protein involved in calcium homeostasis, where it disrupts normal organelle function by reducing the mitochondrial membrane potential and increasing both intracellular Ca²⁺ and ROS levels (Lu *et al.,* 2024). In parallel, Hcp2b targets the mitochondria by binding to SLC25A4 (also known as ANC1 or ANT1), an ADP/ATP transporter and key regulator of the mitochondrial permeability transition pore (MPTP), inducing mitochondrial damage (Lu *et al.,* 2025). 

Interestingly, a manganese-containing catalase, KatN, has been described as a T6SS substrate in EHEC, playing a crucial role in neutralizing host oxidative stress. Upon phagocytosis by macrophages, KatN is translocated into the host cytosol, where it decreases intracellular ROS levels, thereby contributing to bacterial survival (Figure 2) (Wan et al., 2017).

*Aeromonas hydrophila*

The T6SS specialized effector VgrG1 contains a C-terminal vegetative insecticidal protein (VIP-2) domain with actin ADP-ribosyltransferase (ADPRT) activity, which disrupts the host actin cytoskeleton (Figure 2). Expression of VgrG1 or its C-terminal domain alone in HeLa cells induces a rounded cell phenotype, followed by apoptosis mediated by caspase-9 activation, reducing cell viability (Suarez et al., 2010). 

Furthermore, another T6SS effector identified in *A. hydrophila* strain AH54 is Rhs2, a rearrangement hotspot (Rhs) protein. The N-terminus of Rhs2 contains a FIX domain, a typical marker of T6SS effectors, and the core region includes a YD-repeat (tyrosine-aspartate) motif, while its C-terminus contains a DNase belonging to the NucA/B superfamily (Wang et al., 2025a). Functional studies demonstrated that Rhs2 acts as a metal ion-dependent DNase toxin, requiring Mn²⁺ or Mg²⁺ for enzymatic activity (Figure 2). Remarkably, this effector exhibits dual-target activity, acting against prokaryotic and eukaryotic cells. Using a *D. discoideum* phagocytosis model, it was shown that Rhs2 plays a crucial role in protecting *A. hydrophila* AH54 from eukaryotic predation (Wang *et al.,* 2025a).

*Burkholderia species*

In *B. pseudomallei* and *B. thailandensis* the T6SS-5 cluster is a key virulence determinant with the specialized effector VgrG5 being crucial to intercellular spread (Toesca et al., 2014; Schwarz et al., 2014; Lennings et al., 2018). VgrG5 contains the gp27/gp5-like domains and a C-terminal extension (CTD) that is highly conserved among isolates of *B. thailandensis, B. pseudomallei, B. mallei*, and *B. oklahomensis*. This domain with unknown function is required for host cell fusion and virulence in *B. thailandensis*, since mutation of the CTD leads to loss of MNGC induction and increased survival of infected mice (Figure 2) (Schwarz *et al.,* 2014). Further studies described the dynamics of host cell-cell fusion during *B. thailandensis* infection, which involves bacteria-induced host cell membrane protrusions, followed by fusion of the membranes of neighboring cells (Kostow and Welch 2022). Using a *vgrG5* mutant strain, it was demonstrated that VgrG5 is required at the membrane fusion step, facilitating bacterial intercellular spread (Kostow and Welch 2022). More studies are required to describe the biological function of the VgrG5 CTD domain in *Burkholderia* species. 

*B. cenocepacia* infection disrupts the actin cytoskeleton in macrophages, forming ‘‘beads on a string’’-like structures. Deletion of AtsR, a negative regulator of virulence factors, leads to T6SS activation and dramatic alterations in macrophage actin cytoskeleton architecture (Aubert et al., 2015). A random transposon mutagenesis screening revealed a non-VgrG T6SS effector responsible for such cytoskeletal changes, named TecA (Aubert *et al.,* 2016). *B. cenocepacia* TecA is a cysteine protease-like hydrolase, that specifically catalyzes asparagine deamidation of Rho GTPases. During infection, TecA can similarly deamidate and inactivate Rac1, which is responsible for *B. cenocepacia*-induced actin cytoskeleton disruption (Figure 2). Also, deamidation of RhoA is responsible for *B. cenocepacia*-stimulated Pyrin inflammasome activation, protecting mice from lethal infection (Aubert *et al.,* 2016). Corroborating this data, it was recently shown that TecA from a clinical strain of *B. cenocepacia* (AU1054) is a virulence factor that exacerbates lung inflammation, weight loss, and lethality in mouse infection models (Loeven et al., 2021).

*Edwardsiella species*

In *Edwardsiella*, a notable example of an anti-eukaryotic effector is EvpP, an effector secreted by different species within the genus. In *E. tarda* strain EIB202*,* EvpP acts by inhibiting NLRP3 inflammasome activation in macrophages and interfering with Ca²⁺- and Jnk-dependent signaling, which promotes bacterial survival and colonization (Figure 2) (Chen et al., 2017). EvpQ is another T6SS effector identified in *E. piscicida* that is translocated into host cells and contributes to pathogenesis (Li et al., 2021). EvpQ shares a conserved domain of C70 family cysteine protease and shares 23.91% identity with the T3SS effector AvrRpt2 of phytopathogenic *Erwinia amylovora*, but its function is still unknown (Li *et al.,* 2021).

*Pantoea ananatis*

A recent study revealed a novel toxic effector in the phytopathogen *P. ananatis* with a eukaryotic-like domain homologous to the human enzyme CD38, an ADP-ribosyl cyclase (ARC) involved in calcium signaling and immune activation. This effector, named ARC^tox^, is a modular protein, harboring an N-terminal PAAR domain and the ARC domain at the C-terminus. Structural analyses revealed that the C-terminal ARC extension adopts a globular fold nearly identical to that of CD38, also conserving the catalytic residues (Martinkus et al., 2025). However, ARC^tox^ functions as an NAD^+^ hydrolase, active on NAD⁺ and NADP⁺ both *in vitro* and *in vivo*, leading to depletion of these cofactors in bacterial cells and subsequent growth inhibition. Cytotoxicity against eukaryotic cells of its C-terminal domain was demonstrated by heterologous expression in the model yeast *S. cerevisiae*, highlighting its broad-spectrum activity (Martinkus *et al.,* 2025). Importantly, the ARC domain is widespread in bacteria, commonly found as a module associated with distinct polymorphic toxins and secretion motifs, including T5 and T7 secretion systems, suggesting a general function as a toxin required for interference competition or host interaction. Further studies are required to investigate whether the anti-eukaryotic activity of ARC^tox^ is deployed for pathogenicity to plants and if it is directly secreted into plant cells. 

*Xanthomonas oryzae*

The T6SS-2 cluster of the plant pathogen *X. oryzae* strain PXO99A is required for bacterial competition both *in vitro* and *in planta*. Two bactericidal phospholipase effectors, TleA and TleB, have been identified. TleB functions as a trans-kingdom effector that mediates interactions with non-hosts plants, modulating plant reproductive development by two independently described mechanisms (Tan et al., 2025; Yang et al., 2025). Tan *et al.* (2025) demonstrated that TleB has lipase activity and interacts with anionic lipids *in vitro*, but its expression in plants was not toxic. In another study, Yang *et al.* (2025) demonstrated that TleB expressed in plant cells interacts with PUB14, a plant E3 ubiquitin ligase involved in ovule initiation and seed formation. Under normal conditions, PUB14 (PLANT U-BOX 14) binds to and stabilizes the transcription factor BZR1 through mono-ubiquitination, a modification that promotes ovule initiation and ensures proper seed formation. TleB competitively binds PUB14, blocking its interaction with BZR1 and consequently decreasing BZR1 stability and abundance, which inhibits seed formation. In parallel, PUB14 also ubiquitinates TleB, targeting it for degradation via the 26S proteasome, suggesting a balance between reproduction and immunity (Yang *et al.,* 2025). Future research may clarify the distinct mechanisms employed by TleB to manipulate reproduction in non-hosts plants. Furthermore, direct secretion of TleB into plant cells remains to be demonstrated.

## T6SS anti-eukaryotic effectors that target fungal cells for niche competition

*S. marcescens* Tfe1 and Tfe2 were the first T6SS effectors identified for their role in fungal competition against *C. albicans* (budding and filamentous form), *C. glabrata*, and *S. cerevisiae* (Trunk et al., 2018). Notably, these effectors target distinct cellular pathways to intoxicate fungi: Tfe1 causes plasma membrane depolarization, whereas Tfe2 causes imbalance of intracellular amino acid levels by affecting uptake and biosynthesis, which results in induction of the autophagy pathway (Figure 3, Table S1) (Trunk *et al.,* 2018). Despite these well-characterized intracellular effects, it remains unclear how T6SS effectors are translocated across the fungal cell walls, representing an important open question in the field.

Further studies on other antifungal T6SSs have identified distinct effectors with nuclease activity. The *A. baumannii* effector TafE (ACX60_15365) is required for T6SS-mediated fungal killing. Toxicity is caused by DNA degradation, which is mediated by a C-terminal Ntox15 domain with a HXXD motif, commonly found in nucleases (Figure 3, Table S1). Interestingly, TafE is specifically recognized by the yeast nuclear import machinery but not by HeLa cells, where it accumulates in the cytoplasm without causing toxicity (Luo et al., 2023). Analysis of TafE distribution among *A. baumannii* isolates demonstrated its presence in a small subset of strains, thereby expanding the activity of a T6SS that otherwise targets bacterial cells to include antifungal functions.

A new DNase, named TseN, has been recently identified as the primary effector mediating the antifungal activity of the *A. citrulli* T6SS against *S. cerevisiae*, *C. neoformans* and distinct *Candida* species, including all *C. auris* clades (Yan et al., 2025). TseN contains two nucleolar localization sequences required for nuclear localization in *S. cerevisiae* cells, where it causes cytotoxicity by promoting DNA fragmentation (Figure 3, Table S1). Notably, *A. citrulli* outcompetes the fungal pathogen *C. auris in vivo*, using a mouse model of skin infection, and TseN was required for the T6SS-mediated fungal killing. TseN is part of a new family of widely distributed nucleases, which lack previously characterized nuclease motifs. Further investigation in other bacterial species may reveal new aspects of this novel anti-eukaryotic effector.

The *L. enzymogenes* Le1893 contains an AHH nuclease domain associated with a PAAR domain and an RHS core, which are commonly found in T6SS effectors. Although not required for the antifungal activity of the T6SS in *L. enzymogenes*, ectopic expression of this effector in yeast cells was toxic and caused reduced genomic DNA abundance, suggestive of nuclease activity (Lin et al., 2025). Further studies are required to characterize the function and cellular targets of this new effector.

We previously mentioned the *K. pneumoniae* trans-kingdom effector VgrG4, which mediates killing of bacteria and fungi, and manipulates the innate immune response in epithelial cells by a mechanism that generates ROS and targets the mitochondria of eukaryotic cells (Figures 2 and 3) (Storey et al., 2020; Sá-Pessoa et al., 2023). Deletion of *vgrG4* abrogated T6SS-mediated killing of *C. albicans* and *S. cerevisiae*, demonstrating that it is the main effector involved in antifungal activity of hypervirulent *K. pneumoniae* (Storey *et al.,* 2020).

A new effector function has been described for TfeC, a *Y. pseudotuberculosis* effector required for T6SS4-mediated fungal killing in this bacterium (Figure 3) (Zhu et al., 2026). Structural and biochemical analyses revealed that TfeC is a novel chitinase that forms a distinct branch within the GH18 family and kills fungal cells by directly targeting the cell wall (Zhu *et al.,* 2026). This exciting finding highlights the functional diversity of T6SS effectors and suggests that additional antifungal activities remain to be discovered. 

## Concluding remarks

Type VI secretion systems have evolved as multifaceted nanomachines that function at the interface between bacteria and eukaryotic cells across environmental and host-associated contexts. Although long considered primarily as weapons for interbacterial competition, it is now clear that many T6SSs directly manipulate eukaryotic biology, contributing to predator resistance, fungal antagonism, host colonization, immune modulation, and tissue dissemination. 

Many of the effectors discussed in this review possess well-characterized enzymatic domains and clearly defined mechanisms of action. However, numerous others play central roles in the manipulation of eukaryotic cells-by disrupting signaling pathways, modulating immune defenses, and altering fundamental cellular processes-yet their precise molecular targets and catalytic activities remain largely undefined. Elucidating these mechanisms will require integrated approaches which will be essential to uncover the full spectrum of anti-eukaryotic strategies deployed by T6SSs. At the same time, the discovery of trans-kingdom effectors underscores the functional plasticity of these systems and highlights the boundaries between environmental adaptation and virulence. 

The identification of novel anti-eukaryotic effectors and their cellular targets may be boosted by the use of *S. cerevisiae* as a model organism for heterologous expression (Bosis et al., 2011). Growth inhibition following endogenous expression in yeast is indicative of an anti-eukaryotic activity, which can be further explored to identify cellular targets, given the extensive genetic toolkit available for yeast and the high degree of conservation of essential cellular processes across eukaryotes. For instance, a high-throughput genetic screening strategy designed in yeast to identify membrane-associated T3SS and T4SS effectors revealed that 30% of the 200 effectors tested were membrane-associated in fungal cells. These findings provided important insights into the functions of many effectors that had previously remained poorly characterized (Weigele et al., 2017). 

For many T6SSs, direct effector delivery into plant or animal cells has yet to be conclusively demonstrated, and the evolutionary links between environmental interactions with amoebae, fungi, and host-associated pathogenicity are still poorly defined. Addressing these gaps will require integrative approaches combining structural biology, advanced cellular models, *in vivo* infection systems, and microbiome-level analyses. A deeper understanding of the role of T6SSs in interactions between bacteria and eukaryotes may reveal new strategies for therapeutic intervention or microbiome modulation. 

## Supplementary material

The following online material is available for this article:

Table S1 - Anti-eukaryotic effectors of T6SSs.

## Data Availability

 Data sharing is not applicable to this article as no new data was generated in this study.
