# Supplementary material for: Type VI secretion systems at the interface between bacteria and eukaryotic cells
Source: Genet Mol Biol. 2026 Sep 14;49(Suppl 1):e20260024. doi: 10.1590/1678-4685-GMB-2026-0024 (PMC13577199; doi:10.1590/1678-4685-GMB-2026-0024)
Supplement: Supplementary file 1 [file 1415-4757-GMB-49-s1-e20260024-s1.pdf]

Genetics and Molecular Biology
https://doi.org/10.1590/1678-4685-GMB-2026-0024

Supplementary Material to "Type VI Secretion Systems at the Interface Between Bacteria and Eukaryotic Cells"

Table S1 – T6SS anti-eukaryotic effectors.

Effector	Cellular Target / Protein Activity	Conserved domain	Host effects	Target / Dual function[1]
Aeromonas hydrophila				
VgrG1	ADP-ribosyltransferase (ADPRT) activity	VIP-2 domain	Disrupts the host actin cytoskeleton and induces apoptosis	HeLa cells / N.D.
Rhs2	Causes DNA fragmentation	Metal ion-dependent DNase	Unknown	Amoebae / Yes
Acinetobacter baumannii				
TafE	Causes DNA fragmentation	DNAse Ntox15 domain	Induces cell death	Yeast / Yes
Acidovorax citrulli				
TseN	Causes DNA fragmentation	DNAse domain	Nucleolar localization, DNA degradation	Yeast / Yes
***Burkholderia* species**				
TecA	Catalyzes asparagine deamidation of Rho GTPases	Deamidase	Host actin cytoskeleton disruption and Pyrin inflammasome activation	Diverse / N.D.
VgrG5	Unknown	Unknown	Cell fusion and multinucleated giant cell formation	Diverse / N.D.
***Edwardsiella* species**				
EvpP (*E. tarda* EIB202)	Unknown	Unknown	Suppresses NLRP3 inflammasome activation	Diverse / N.D.
EvpQ	Unknown	C70 family cysteine protease	Unknown	Fish / N.D.
Escherichia coli				

Effector	Cellular Target / Protein Activity	Conserved domain	Host effects	Target / Dual function[1]
Hcp2b	Interacts which ADP/ATP transporter carrier (SLC25A4) a mitochondria-associated protein	Unknown	Induces mitochondrial damage	Bird / N.D.
Hcp2a	Interacts with the mitochondrial protein Leucine zipper EF-hand-containing transmembrane protein 1 (LETM1)	Unknown	Reduces mitochondrial membrane potential; increases mitochondrial calcium and ROS levels.	Bird / N.D.
KatN	Detoxifies intracellular ROS	Catalase	Improves bacterial survival in host cells	Macrophage / No
Hcp1 (EPEC strain)	Unknown	Unknown	Promotes acute kidney and liver injuries in mice; triggers pyroptosis through NLRP3 inflammasome activation.	Diverse / N.D.
Hcp1 (K1 strain)	Unknown	Unknown	Induces actin cytoskeleton rearrangement and triggers caspase-8–dependent apoptosis	HBMEC/ N.D.
***Francisella* species**				
PdpC	Unknown	Unknown	Facilitates phagolysosomal escape, intracellular replication, and efficient erythrocyte invasion	Macrophage / No
PdpD	Unknown	Unknown	Contributes to phagosome rupture and activates AIM2 inflammasome	Mice / N.D.
OpiA	Generates phosphatidylinositol-(3)-phosphate on endosomes	Phosphatidylinositol 3-kinase	Facilitates phagosomal escape in infected host cells	Macrophage / N.D.
OpiB	Unknown	CTD ankyrin repeat and NTD conserved cysteine protease	Contributes to intracellular growth in phagocytic cells	Macrophage / N.D.
Klebsiella pneumoniae				
VgrG4	Induces Ca2+ influx from the ER to the mitochondria	DUF2345 ROS toxic (RTD)	Mitochondrial fragmentation, cytoskeleton rearrangements, and activation of the NLRX1 receptor to produce ROS	Diverse / Yes
Lysobacter enzymogenes				
Le1893	Unknown	RHS and AHH nuclease superfamily	Inhibits hyphal growth, reduction of yeast genomic DNA abundance	Filamentous fungi / N.D.
Myxococcus xanthus				
MXAN_RS08765	Unknown	Unknown	Induces cell death	*S. cerevisiae* / Yes
Pseudomonas aeruginosa				
PldA/Tle5a	Binding to Akt kinase	Phospholipase D3	Activation of PI3K/Akt pathway leading to actin rearrangement and protrusion formation	Epithelial / Yes

Effector	Cellular Target / Protein Activity	Conserved domain	Host effects	Target / Dual function[1]
PldB/Tle5b	Binding to Akt kinase	Phospholipase D	Activation of PI3K/Akt pathway leading to actin rearrangement and protrusion formation	Epithelial / Yes
VgrG2b	C-terminal fragment competes with NEK7 to bind to NLRP3	Zn-dependent metallopeptidase; DUF2345	Interacts with γ-tubulin and inhibits NLRP3 inflammasome activation	Diverse / Yes
TplE	Disruption of the ER compartment	PGAP1-like	Induces the unfolded protein response and autophagy	Diverse / Yes
$TseL^{PA}$	Unknown	Lipase	Promotes bacterial internalization	Diverse / Yes
Yersinia pseudotuberculosis				
TseR	Degradation of preferentially single-stranded RNA	Ntox44 CTD	Facilitates virulence and colonization	Epithelial / N.D.
TkeA	Causes DNA damage in mammalian cells	GIY-YIG nuclease	Activates the cGAS-STING pathway and induces apoptosis	Epithelial / N.D.
TfeC	Chitinase targeting the fungal cell wall	GH18 family of chitinases	Damages the fungal cell wall	Fungal / N.D.
TssS	Mn2+ chelating protein	Unknown	Inhibits activation of the cGAS-STING pathway by chelating Mn^{2+}	HeLa cells / N.D.
***Vibrio* species**				
VgrG-1	Covalently crosslinks monomeric G-actin	ACD CTD	Depolymerization of actin stress fibers	Diverse / No
VasX	Pore formation	NTD pleckstrin homology (PH); CTD colicin; MIX-domain	Membrane disruption through pore formation	Amoeba / Yes
TseL	Unknown	Phospholipase domain	Unknown	Amoeba / Yes
Tie1/2	Unknown	Unknown	Induction of pyroptotic cell death	Macrophage / No
Vpr01570	Unknown	CNF1 toxin domain; MIX domain	Actin rearrangement	Diverse / No
Pantoea ananatis				
ARC^{tox}	Hydrolyzes NAD^{+} and $NADP^{+}$	ADP ribosyl cyclase	Inhibition of cellular growth	Yeast / Yes
Serratia				
Tfe1	Unknown	Unknown	Membrane depolarization leading to loss of membrane integrity	Yeast / N.D.
Tfe2	Unknown	Unknown	Nutrient imbalance, impairment of membrane transporters activity, and autophagy induction	Yeast / No
Xanthomonas oryzae				
TleB	Interaction with E3 ubiquitin ligase UB14 and binds to anionic phospholipids	Phospholipase	Impaired ovule initiation	Plant / Yes

1- Dual function: effect in both prokaryotic and eukaryotic cells; N.D. - Not determined; CTD – C-terminal domain; NTD – N-terminal domain; HBMEC- Human brain microvascular endothelial cells; ACD - actin cross-linking domain; ER - Endoplasmic reticulum; ROS - Reactive oxygen species. References are provided in the main text.
